# Part II: Can School‐Based Delivery of an Evidence‐Based Parenting Program Promote the Home–School Partnership? Parental Self‐Regulation as the Mechanism of Change

**DOI:** 10.1111/famp.70150

**Published:** 2026-04-28

**Authors:** Tianyi Ma, Cassandra L. Tellegen, Julie Hodges, Christopher Boyle, Matthew R. Sanders

**Affiliations:** ^1^ Parenting and Family Support Centre, School of Psychology The University of Queensland Brisbane Australia; ^2^ School of Education The University of Adelaide Adelaide Australia

**Keywords:** cluster randomized trial, family–school partnership, mechanism of change, parent engagement, parent involvement, parental self‐regulation, triple P

## Abstract

High‐quality partnerships between families and schools can bring enormous benefits to the development, learning, and well‐being of children. Although past literature has frequently identified the parenting self‐efficacy element of parental self‐regulation as a key determinant of the strength of the home–school partnership, this knowledge base relies heavily on cross‐sectional studies. This paper follows on from an article in *Family Process* reporting the findings of a cluster randomized trial of an evidence‐based parenting program designed to promote parental self‐regulation, namely the seminar version of the Triple P—Positive Parenting Program. The trial found post‐intervention improvements in two domains of the home–school partnership, namely parent–teacher communication and parent school‐based involvement. Given that the home–school partnership was not a target of the intervention, the current study conducted a mechanism of change analysis to examine whether improvements in the home–school partnership could be attributed to post‐intervention improvements in parental self‐regulation. Data were collected from a sample of 912 parents of children attending 160 different primary schools across three Australian states. Following a Random Intercept Cross‐lagged Panel Model (RI‐CLPM) approach, bidirectional within‐participant effects between parental self‐regulation and parent–teacher communication were found, while unidirectional within‐participant effects were found for parent school‐based involvement. The findings provided support to the proposed theory of change where improvements in parental self‐regulation are the underlying mechanism behind reported post‐intervention improvements in the quality of the home–school partnership. The findings also support the potential spillover benefits of school‐based delivery of evidence‐based parenting programs.

**Trial Registration:** Australian New Zealand Clinical Trials Registry: ACTRN12623000852651

## 1 Introduction

Following the transition to formal schooling, families and schools become two of the most influential environments that shape children's development, learning, and well‐being. Represented by Bronfenbrenner's meso‐system level (Bronfenbrenner and Morris 2006), families and schools tend to form relationships with and exert influence on each other. In this study, the term *home–school partnership* refers to the interaction between families and schools. Bierman and Sheridan (2022) define it as a multidimensional construct encompassing contributions from parents, teachers, and schools, as well as the dynamic quality of parent–teacher communication and relationships. This concept serves as an umbrella term that includes related constructs such as family–school partnership, parent involvement, and parent engagement (Bierman and Sheridan 2022). Key dimensions of the home–school partnership include home‐based involvement (e.g., supporting learning at home, discussing school experiences with children), school‐based involvement (e.g., attending parent–teacher meetings, volunteering), parental expectations and aspirations for children's education, and parent–teacher communication and relationships (Barger et al. 2019; Wilder 2014). There is growing recognition that this partnership operates bidirectionally, with mutual influence between families and schools (Bierman and Sheridan 2022).

### 1.1 The Value of Effective Home–School Partnership

Extensive research has shown that high‐quality home–school partnerships significantly benefit children's learning. A systematic review of 75 studies (Boonk et al. 2018) identified four key parental contributions linked to better learning outcomes: reading at home, high expectations, frequent communication about school, and praising effort and performance. Meta‐analyses consistently show a positive relationship between all dimensions of the home–school partnership and academic achievement. Wilder's (2014) umbrella meta‐analysis of nine meta‐analyses confirmed this association across definitional variations. A large‐scale meta‐analysis of 448 studies with 480,830 families conducted by Barger et al. (2019) found that home‐based involvement, school participation, and parent–teacher communication, but not homework assistance, were positively associated with academic achievement (*r* = 0.13), engagement (*r* = 0.15), and motivation (*r* = 0.23). Similarly, an umbrella meta‐analysis of 1177 studies by Kim (2022) found all parental contributions, except homework assistance, were linked to improved learning outcomes (*r* = 0.18). It is argued that high‐quality home–school partnerships enable parents to be more effective at building children's cognitive capacity and socio‐emotional skills when they know what they can do to support children and the expectations from the schools (Barger et al. 2019). Also, communications between families and schools enable the accurate exchange of information about children's progress and needs, enabling both parties to be more responsive in optimizing skill acquisition and addressing challenges (Barger et al. 2019).

Beyond academics, home–school partnerships also support children's social, emotional, and behavioral well‐being. Barger et al. (2019) found the quality of the home–school partnership had positive associations with children's social (*r* = 0.12) and emotional adjustment (*r* = 0.17) and a negative association with behavioral problems in children (*r* = −0.15). Additionally, a meta‐analysis of 77 studies evaluating home–school partnership interventions showed structured parental involvement programs improved children's social competence (*g* = 0.32) and mental health (*g* = 0.34; Sheridan et al. 2019). Across meta‐analyses, the majority of the included studies were conducted in the United States using cross‐sectional designs, with small numbers of international studies and longitudinal studies (Barger et al. 2019; Kim 2022; Sheridan et al. 2019). In the Australian context, beyond parent's involvement at home, home–school partnership typically includes one or two parent–teacher conferences per year, school‐based events, and informal communication with teachers. However, national data suggest that Australian parents report lower levels of engagement in school‐based activities compared to the average of 38 member countries of the Organisation for Economic Co‐operation and Development (2023), highlighting potential scope for strengthening home–school partnerships.

### 1.2 Parenting Self‐Efficacy, Parental Self‐Regulation, and the Home–School Partnership

One of the most frequently studied parent‐level antecedents to parents' contributions to the home–school partnership is parenting self‐efficacy, which is adapted from Bandura's (1997) Social Cognitive Theory and defined as parents' confidence in their ability to promote child development and other desirable outcomes through changing their own behaviors (Albanese et al. 2019; Fang et al. 2021; Sanders and Mazzucchelli 2013). Its positive associations with the home–school partnership have been consistently found in cross‐sectional studies (e.g., Ferretti et al. 2019; Ice and Hoover‐Dempsey 2010; Newland et al. 2013), which evaluated influential theoretical models and frameworks of home–school partnership (e.g., Hoover‐Dempsey and Sandler 1997; Hoover‐Dempsey et al. 2005). An overall positive association between parenting self‐efficacy and parents' contributions to the home–school partnership (*r* = 0.19) was found in a recent meta‐analysis which synthesized findings from 50 independent studies involving over 20,000 primary school‐aged children (Ma, Tellegen, Hodges, and Sanders 2024). In addition, the positive associations have been identified in recent large‐scale longitudinal studies that controlled for the dynamic changes in each variable (Choe 2022; Ma, Tellegen, and Sanders 2024).

Recent developments in parenting self‐efficacy research have moved toward a focus on the broader construct of parental self‐regulation. This extends Bandura's (1997) work and is defined as parents' capacity to self‐monitor, to adapt their own attributions and behavior, as well as to be independent problem‐solvers in their parenting role (Sanders and Mazzucchelli 2013; Sanders et al. 2019). The parental self‐regulation framework views parenting self‐efficacy as one of five core elements (Sanders and Mazzucchelli 2013), with the other four elements being: self‐sufficiency—having sufficient knowledge and skills to be an effective parent; self‐management—being able to improve the parenting practices; personal agency—attributing positive changes in children to their own behavior and effort; and problem‐solving—being able to develop and execute a plan to solve a defined parenting problem. While the present evidence on parental self‐regulation and its relationship to the home–school partnership primarily has focused on parenting self‐efficacy (Ma, Tellegen, Hodges, and Sanders 2024), recent measurement development studies found that parenting self‐efficacy was a strong indicator of the overall self‐regulatory capacity of parents (*r* = ~0.70, factor loading = 0.94; Hamilton et al. 2015; Tellegen et al. 2022). One recent Australian study with over 2000 parents suggested that the included measure of parental self‐regulation displayed stronger positive associations with various aspects of the home–school partnership than the measure of parenting self‐efficacy (Ma et al. 2026b). More research on parental self‐regulation is urgently needed.

### 1.3 Evidence‐Based Parenting Programs

Knowledge gaps remain in understanding the associations between parental self‐regulation and the quality of the home–school partnership, as well as the direction of influence. One major way of promoting parental self‐regulation is through the provision of evidence‐based parenting programs (Moller et al. 2024; Sanders et al. 2021; Sanders and Mazzucchelli 2013; Sanders, Turner, et al. 2024). Therefore, as parental self‐regulation is a key program target promoted through program participation, randomized controlled evaluations of evidence‐based parenting programs provide an excellent opportunity to experimentally explore the associations between parental self‐regulation and the home–school partnership (Ma, Tellegen, Hodges, and Sanders 2024).

One system of evidence‐based parenting programs is the Triple P—Positive Parenting Program, which is one of the most extensively studied and widely disseminated parenting programs that have demonstrated its effectiveness in promoting parental self‐regulation, parenting practices, and a wide range of child outcomes (Sanders et al. 2014). Triple P is a system of intervention with programs varying in their intensity to accommodate the needs and preferences of different families. Low‐intensity variants, such as the seminar intervention, have been found to be able to improve parental self‐regulation, parenting practices, parental adjustment, and children's social, emotional, and behavioral adjustment with small to medium effects (Boyle et al. 2026; Sanders et al. 2009, 2014). Part I of this article series reports the findings of a stepped wedge cluster randomized trial of a series of Triple P seminars delivered through Australian primary schools on home–school partnership (Ma et al. 2026a). Improvements in parent–teacher communication and parent school‐based involvement were found following the intervention with small effect sizes. Positive findings on the home–school partnership were also found in a recent qualitative study of the school staff involved in the trial (Hidajat et al. 2026). Given that these are not the targeted outcomes of the intervention, post‐intervention improvements in parental self‐regulation have been proposed to be the potential mechanism of change.

### 1.4 The Present Study

Following on from the previous findings of post‐intervention improvements in parent–teacher communication and parent school‐based involvement identified in a recent school‐based cluster randomized trial of Triple P seminars (Boyle et al. 2023, 2026; Ma et al. 2026a), the aim of the present exploratory study was to explore whether parental self‐regulation was the mechanism of change. This analysis could also shed light on the direction of the associations between home–school partnership and parental self‐regulation.

## 2 Method

### 2.1 Participants

The sample included 912 parents of children attending 160 primary schools from three Australian states: Queensland (39.8%), South Australia (9.6%), and Victoria (50.5%). The size of the schools varied considerably from less than 25 enrolments to over 3000 enrolments, and the number of parents recruited from each school ranged from one to 45 (*M* = 5.70, SD = 6.59). The majority of parents identified themselves as the primary caregiver (93.2%), mother (85.3%), married or cohabiting (85.9%), held university degrees (76.7%), and were employed (84.8%). Child gender was evenly distributed, and age was largely balanced across the range, though fewer children were in higher grades. Most children attended public schools (81.6%) with over 500 students (64.0%), located in major cities (82.6%), and from above‐average socioeconomic backgrounds (90.2%). School socioeconomic status was captured by the index of community socio‐educational advantage (ICSEA), which is a composite measure developed by the Australian Curriculum, Assessment and Reporting Authority to summarize the socio‐educational backgrounds of students at a school based on factors such as parental education, parental occupation. Further demographic details are in Table 1.

**TABLE 1:** Family, children, and school demographics for both subsamples.

Factor name	*N* (%)	Factor name	*N* (%)
**Relationship to the child**		**Child gender**	
Mother	778 (85.3)	Male	451 (49.5)
Father	124 (13.6)	Female	457 (50.2)
Others	10 (1.1)	Other/prefer not to say	3 (0.3)
**Main caregiver**		**Child age**	
Yes	848 (93.2)	4	13 (1.4)
No	62 (6.8)	5	115 (12.6)
**Another important caregiver**		6	149 (16.3)
Present	853 (93.6)	7	151 (16.6)
Absent	58 (6.4)	8	134 (14.7)
**Household composition**		9	106 (11.6)
Original family	760 (83.4)	10	119 (13.0)
Stepfamily	23 (2.5)	11	84 (9.2)
Sole parent/family	90 (9.9)	12	34 (3.7)
Others	38 (4.2)	13	6 (0.7)
**Language spoken at home**		**State**	
English only	642 (70.4)	Queensland	363 (39.8)
Other languages	270 (29.6)	South Australia	88 (9.6)
**Indigenous Australians**		Victoria	461 (50.5)
No	904 (99.1)	**School sector**	
Yes	8 (0.9)	Public	744 (81.6)
**Marital status**		Catholic	108 (11.8)
Married	680 (74.6)	Independent	60 (6.6)
Cohabiting/de Facto	103 (11.3)	**School size**	
Divorced/separated	65 (7.1)	Less than 100 enrolments	26 (2.9)
Single	53 (5.8)	100–499 enrolments	302 (33.1)
Widow/er	7 (0.8)	500–999 enrolments	459 (50.3)
Others	4 (0.4)	More than 1000	125 (13.7)
**Parental education**		**School SES percentile**	
Less than year 10	1 (0.1)	0%–25%	21 (2.3)
Year 10–11	17 (1.9)	26%–50%	68 (7.5)
High school completion	42 (4.6)	51%–75%	221 (24.2)
Trade/Apprenticeship	5 (0.5)	76%–100%	602 (66.0)
TAFE/College Certificate	148 (16.2)	**School location**	
Undergraduate degree	331 (36.3)	Major cities	753 (82.6)
Postgraduate degree	368 (40.4)	Inner regional	110 (12.1)
**Employment**		Outer regional	40 (4.4)
Employed full time	432 (47.4)	Remote	9 (1.0)
Employed part‐time	281 (30.8)	Very remote	—
Employed casually	60 (6.6)	**Intervention attendance**	
On maternity leave	14 (1.5)	0 session	253 (27.7)
Full‐time student	10 (1.1)	1 session	196 (21.5)
Looking for work	32 (3.5)	2 sessions	179 (19.6)
Not in paid employment	83 (9.1)	3 sessions	284 (31.1)

*Note:* Due to missing data, the sum of participants may not add up to the total.

### 2.2 Design and Procedure

This study is conducted in the context of a stepped wedge cluster randomized trial of Triple P Seminars in Australian primary schools. The trial was registered with the Australian New Zealand Clinical Trials Registry and was conducted in strict accordance with the published protocol (Boyle et al. 2023). Ethics approvals were obtained from the University of Queensland Human Research Ethics Committee (ID: 2022/HE001114), the University of Adelaide Human Research Ethics Committee (ID: 37018), Monash University Human Research Ethics Committee (ID: 36385), and the appropriate education authorities. All processes and procedures were developed in accordance with the 1964 Helsinki declaration and its later amendments or comparable ethical standards as well. Informed consents were collected from all parents.

All principals of primary schools in Queensland, South Australia, and Victoria that we received ethical clearance to contact were provided with information about this project. As discussed in detail in papers reporting the primary findings of this cluster randomized trial (Boyle et al. 2026; Ma et al. 2026a), a total of 380 schools expressed interest in participating and were randomized to batches and steps. Our project team sent promotional materials to schools to distribute to parents at the scheduled intervention delivery time. Participation was voluntary, and parents enrolled using the links provided on the promotional materials. We received 2265 parent registrations from 197 schools to participate in this evaluation. After accounting for missing data, the final sample consisted of 912 parents from 160 schools. The same sample from the main cluster randomized trial papers was used in the present study. The engagement data must be interpreted within the context of a cluster randomized trial of a low‐intensity seminar intervention where financial reimbursement was not available for the post‐intervention and follow‐up questionnaires due to funding constraints. To evaluate the intervention, online parent surveys were administered through the Qualtrics platform three times: at parent registration (baseline, T1), 6 weeks following registration (post‐intervention, T2), and 12 weeks following registration (follow‐up, T3). Under this trial design, all parents who enrolled in the project received the same intervention and had a 6‐week period between T1 and T2 assessments to sign up for and attend seminars online via videoconferencing software individually. To enhance accessibility, each seminar topic was offered at multiple times throughout the intervention window. Both the study design and the CONSORT diagrams were provided in the main trial papers (Boyle et al. 2026; Ma et al. 2026a).

### 2.3 Intervention

As described briefly in the introduction, the low‐intensity intervention consists of a series of three interconnected Triple P seminars, covering the following topics: “The Power of Positive Parenting” (first seminar of the Select Triple P program; Sanders et al. 2009), “Helping Your Child to Manage Anxiety” (seminar version of the Fear‐less Triple P program; Cobham et al. 2017), and “Keeping your Child Safe from Bullying” (seminar version of the Resilience Triple P program; Healy and Sanders 2014). These seminars are designed to improve the self‐regulatory capacity of parents, which could potentially, in turn, enhance the quality of the home–school partnership.

### 2.4 Measures

#### 2.4.1 Home–School Partnership

The quality of the home–school partnership was assessed using the 18‐item, parent‐reported Partners in Education Survey (PIES; Johnson et al. 2025; Kirby et al. 2024). PIES includes five subscales (3–5 items each) that evaluate key aspects of home–school collaboration: Parent–Teacher Communication (e.g., “I feel comfortable talking to my child's teacher”), Parent–School Relationship (e.g., “My child's school makes me feel like an important partner in my child's education”), Home Involvement (e.g., “I talk to my child about their day at school”), Parental‐School Involvement (e.g., “I help out with activities outside the classroom, such as tuckshop or school events”), and Working with the Community (e.g., “My child's school is actively involved in the community and local events”). Parents rated each item on a 10‐point Likert scale (1 = *never/not at all* to 10 = *always/very much*). Subscale scores were calculated as the average of their respective items, with higher scores indicating more positive perceptions of the home–school partnership. All subscales demonstrated strong internal consistency across three assessments (Cronbach's *α* = 0.80–0.92).

#### 2.4.2 Parental Self‐Regulation

Parent's self‐regulatory capacity in their parenting role was measured by the 12‐item, parent‐report measure, Parenting Self‐Regulation Scale (PSRS; Tellegen et al. 2022). Example items include “I have the skills to be an effective parent.” and “I can apply what I learn about parenting solutions to different situations.” Each item was rated on a 7‐point Likert scale from 1 (*strongly disagree*) to 7 (*strongly agree*). A total score is calculated by averaging all items, with higher scores reflecting higher parental self‐regulation. The PSRS demonstrated excellent internal consistency across three assessment points (Cronbach's *α* range from 0.91 to 0.93).

### 2.5 Analytic Plan

Following missing data analysis, the expectation maximization algorithm (EM) was used to impute missing values to preserve statistical power and provide unbiased estimates of intervention effects (Bennett 2001). As parents were recruited through schools, we estimated the intra‐cluster correlation (ICC) and the design effect (DEFF) coefficients to determine the level of analysis. Results suggested that both the ICC and the DEFF were considerably lower than the recommended cutoffs of ICC < 0.10 and DEFF < 1.5, where meeting these cutoffs indicated relatively low risk of biased standard error estimation in single‐level analysis (Huang 2018; Lai and Kwok 2014; Peugh 2010). The average ICC was 0.03 and the average DEFF was 1.16, with no measure at any time‐point exceeding these cutoffs. Therefore, the application of the single‐level approach in the mechanism of change analysis is warranted as the focus of these analyses was at the individual level and with the low ICC and DEFF values.

To evaluate the proposed mechanism of change, where increases in parental self‐regulation following the intervention contributed to the improvements in the home–school partnership, mechanism of change analysis was conducted on home–school partnership outcomes that showed statistically significant intervention effects using *MPlus 7.13* (Muthén and Muthén 2012). The mechanism of change was evaluated through, first, evaluating a Bivariate‐Latent Growth Curve Model (Bivariate‐LGCM; a.k.a. parallel process model), which enabled the associations between post‐intervention changes in parental self‐regulation and changes in the home–school partnership outcome (i.e., correlations between the slope factors) to be explored. Then it was followed by evaluating a Random Intercept Cross‐Lagged Panel Model (RI‐CLPM). With three waves of data available, RI‐CLPM is preferred over other statistical approaches including the bivariate‐LGCM for its strengths in accounting for the dynamic, temporal changes between variables in psychotherapy mechanism of change research, especially in the presence of inertia in variables and bidirectional effects (Falkenstrom 2024; Falkenstrom et al. 2022; Orth et al. 2021; Zyphur et al. 2020). Also, RI‐CLPM separated between‐participant variations and within‐participant variations over time. Within‐participant analysis provides stronger evidence to infer potential direction of the relationships (Falkenstrom 2024; Falkenstrom et al. 2022). We applied *p* < 0.05 as the significance level for between slope associations and cross‐lagged regressions. We followed the recommendations of Falkenstrom et al.'s (2022) to interpret the within‐participant effects. To aid interpretation of cross‐lagged regression coefficients, Orth et al. (2024) proposed the following benchmarks: 0.03, 0.07, and 0.12 for small, medium, and large cross‐lagged effects, respectively. Consistent with the study's focus on longitudinal mechanisms of change, instead of predictors of baseline levels of functioning, demographic covariates were not included in the bivariate‐LGCM or RI‐CLPM.

For both Bivariate‐LGCM and RI‐CLPM analyses, model fit was evaluated using a combination of the chi‐square goodness of fit test (*χ* ^2^), the comparative fit index (CFI), the Tucker–Lewis index (TLI), the root mean squared error of approximation (RMSEA), and the standardized root mean squared residual (SRMR) following guidelines for suggested cutoff values (Byrne 2010). The ideal cutoff values are *p* > 0.05 for *χ* ^2^, CFI > 0.95, TLI > 0.95, RMSEA < 0.05, and SRMR < 0.08. However, models showing CFI > 0.90, TLI > 0.90, and RMSEA < 0.08 are acceptable (Byrne 2010). In addition, *χ* ^2^ goodness of fit tests tend to be overly sensitive to large sample sizes; thus, it is typical to find significant *χ* ^2^ tests in psychology research (Byrne 2010).

## 3 Results

### 3.1 Missing Data Analysis and Data Imputation

Since this study is part of a larger research project, missing data analysis and imputation were conducted using the full dataset rather than the specific dataset used in the present study. To ensure accuracy in data imputation for Intention‐to‐Treat (ITT) analysis, at least 50% data availability was required (Hirose et al. 2016). Consequently, of the 2265 parents who completed the baseline survey, 1632 were excluded for not completing either of the follow‐up surveys, leaving participants who met the minimum data threshold. Comparisons at T1 indicated that nonparticipants were largely similar to participants in terms of demographic factors and outcome measures. However, participants tended to report less consistent parenting practices, lower parent–school relationship quality, and lower parental involvement in school‐based activities. They were also more likely to have attended intervention sessions. In the final sample, missing data percentages were 0.65% at baseline, 6.53% post‐intervention, and 43.41% at follow‐up. No significant associations were found between missing data and demographic or baseline outcome variables. However, Little's MCAR test was significant (*χ* ^2^(120,649, *N* = 912) = 126237.08, *p* < 0.001), indicating that data were not missing completely at random (MCAR). Separate variance *t*‐tests suggested the data were missing at random (MAR). To create a comprehensive dataset, missing values were imputed using the expectation–maximization (EM) algorithm, a widely recommended method for handling and imputing data under the MAR condition (Bennett 2001; Hirose et al. 2016; Schlomer et al. 2010). A final sample of 912 parents was used in all analyses.

### 3.2 Intervention Effects

Intervention effects were evaluated in Part I of this article series (Ma et al. 2026a), and summarized in Table 2. Cohen's *d* effect sizes were reported to aid the interpretation of the magnitude of the effects. Significant improvements in parent–teacher communication and parents' involvement in school‐based activities were found at post‐intervention and maintained at follow‐up assessment, with small effect sizes. Parental self‐regulation was found to improve at post‐intervention with a small to medium effect size, which was further improved to a medium effect size at follow‐up assessment.

**TABLE 2:** Intervention effects and model fit indices for the mechanism of change analysis.

Measure		T1	T2	T3	ES		ES		Intercept (*I*_b_)	Slope (*S*_b_)	*p* for *S*_b_	
		*M* (SD)	*M* (SD)	*M* (SD)	T1–T2		T1–T3					
PIES—home involvement		8.82 (1.34)	8.95 (1.27)	8.88 (1.16)	0.12*		0.04		8.87	0.03	0.218	
PIES—parent–teacher communication		7.57 (2.02)	7.84 (1.96)	7.83 (1.83)	0.16*		0.15*		7.65	0.13	< 0.001	
PIES—parent–school relationship		8.44 (1.66)	8.36 (1.76)	8.32 (1.63)	−0.05		−0.08		8.47	−0.07	0.025	
PIES—parental–school involvement		5.99 (2.47)	6.36 (2.46)	6.43 (2.38)	0.19*		0.22*		6.08	0.22	< 0.001	
PIES—working with the community		7.40 (2.01)	7.59 (2.06)	7.56 (1.95)	0.11*		0.09		7.46	0.08	0.018	
Parenting Self‐regulation Scale		4.86 (0.93)	5.14 (0.93)	5.25 (0.91)	0.35*		0.49*		4.89	0.19	< 0.001	

Model fit indices for the mechanism of change analysis												
Measures	Bivariate‐LGCM						RI‐CLPM					
	*χ* ^2^	CFI	TLI	SRMR	RMSEA	RMSEA [90% CI]	*χ* ^2^	CFI	TLI	SRMR	RMSEA	RMSEA [90% CI]
PIES—PTC ~ PSRS	24.80*	0.992	0.970	0.020	0.076	[0.049–0.105]	15.87	0.996	0.988	0.032	0.049	[0.023–0.077]
PIES—PSI ~ PSRS	32.91*	0.989	0.959	0.027	0.089	[0.062–0.118]	9.72	0.998	0.995	0.029	0.032	[0.000–0.062]

*Note:* Intention‐to‐treat analysis sample was used *N* = 912. *M* and SD = observed means and standard deviations; ES = Cohen's *d* effect sizes based on observed means and standard deviations. Effect sizes were produced by repeated‐measure *t*‐tests for the given time point comparing to T1. Significant intervention effects were determined by significant *p* values for the slope factor (*p* < 0.001). The intervention effects table is a replication of table displays in Part I of this article series (Ma et al. 2026a). Details of the statistical procedure are reported in the cited article.

Abbreviations: Bivariate‐LGCM, bivariate‐latent growth curve model; PIES, Partners in Education Survey; PSRS, Parenting Self‐regulation Scale; RI‐CLPM, Random Intercept Cross‐Lagged Panel Model.

* *p* < 0.001.

### 3.3 Mechanism of Change Analysis

As the intervention was delivered at the individual level, we examined the post‐intervention growth in parental self‐regulation as the underlying mechanism of change at the individual level behind the two aspects of the home–school partnership for which significant positive associations were found: improvements in parent–teacher communication and parent school‐based involvement through bivariate‐LGCMs and RI‐CLPMs. Model fit indices are reported in Table 2.

#### 3.3.1 Parent–Teacher Communication

##### 3.3.1.1 Bivariate‐LGCMs

**FIGURE 1:**
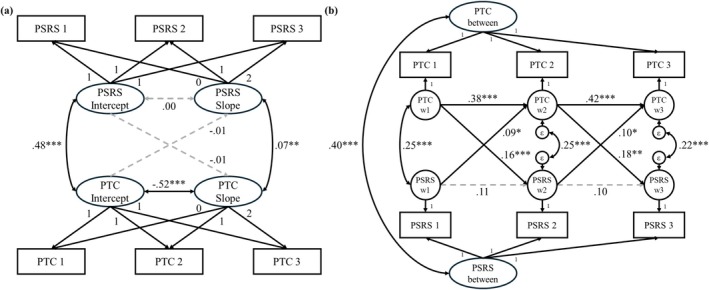
Associations between parental self‐regulation and parent–teacher communication. (a) Bivariate‐Latent Growth Curve Model (LGCM) on parental self‐regulation and parent–teacher communication. Unstandardized estimates are displayed. (b) Random Intercept Cross‐Lagged Panel Model (RI‐CLPM) on parental self‐regulation and parent–teacher communication. Standardized estimates are displayed. between, between‐participant effects; PSRS, Parenting Self‐regulation Scale; PTC, Partners in Education Survey (PIES) Parent–Teacher Communication subscale; w, within‐participant effects. *p < 0.05; **p < 0.01; ***p < 0.001.

As shown in Figure 1a, besides the positive correlation between the intercepts, the slopes of parent–teacher communication and parental self‐regulation were positively associated. This suggested that improvements in parental self‐regulation were positively correlated with improvements in parent–teacher communication. Neither of the intercepts was associated with the slope of the other factor, which suggested that the rate of change in one factor was not associated with the baseline levels of the other factor.

##### 3.3.1.2 RI‐CLPM

As displayed in Figure 1b, the between‐participant effects on parent–teacher communication and parental self‐regulation were positively correlated. Within‐participant analyses revealed that after accounting for the within participant concurrent correlations and autoregressive regressions, parental self‐regulation in early measurement points was positively associated with parent–teacher communication at successive measurement points with a medium to large cross‐lagged regression effect. Parent–teacher communication at earlier measurement points could also positively predict parental self‐regulation at successive measurement points, with a large cross‐lagged regression effect. These bidirectional, within‐participant cross‐lagged effects suggested that improvements in parent–teacher communication could be driven by improvements in parental self‐regulation following the intervention, and vice versa.

#### 3.3.2 Parent School‐Based Involvement

##### 3.3.2.1 Bivariate‐LGCMs

**FIGURE 2:**
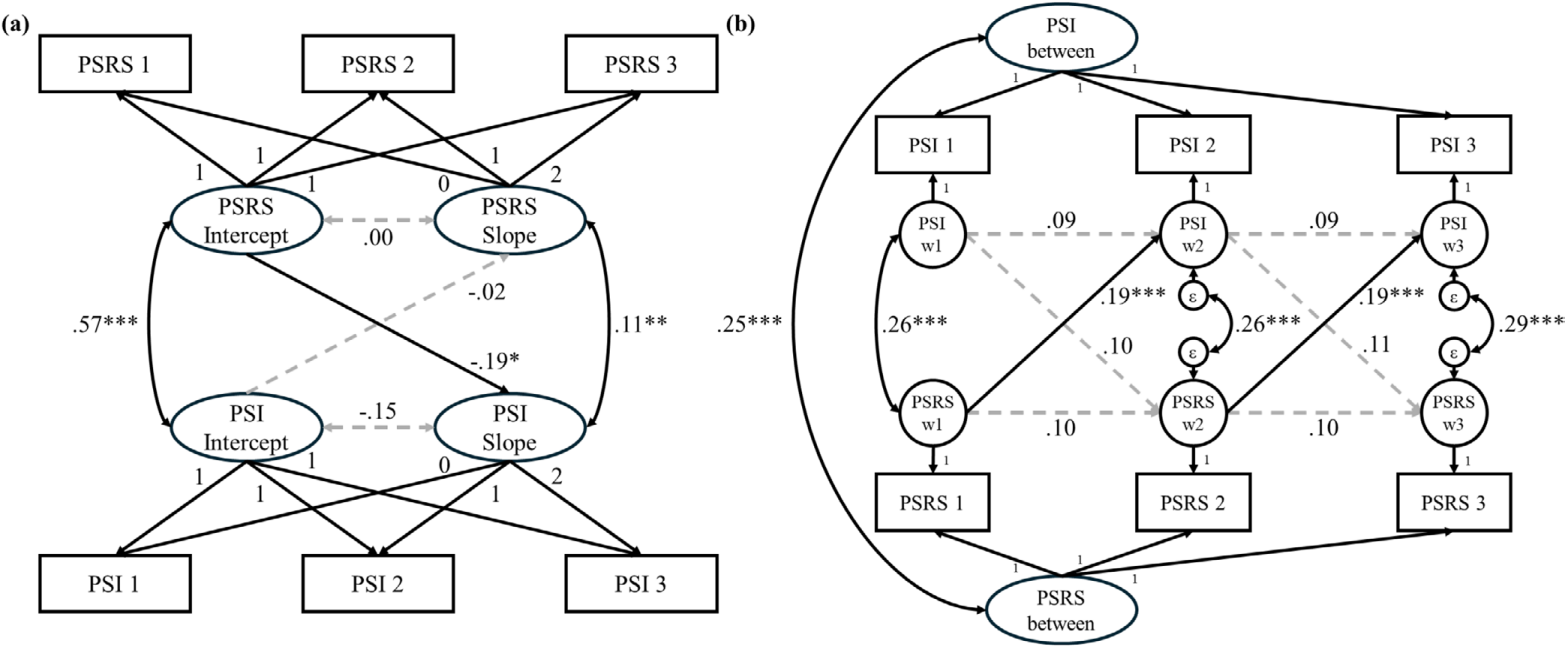
Associations between parental self‐regulation and parent school‐based involvement. (a) Bivariate‐Latent Growth Curve Model (LGCM) on parental self‐regulation and parent school‐based involvement. Unstandardized estimates are displayed. (b) Random Intercept Cross‐Lagged Panel Model (RI‐CLPM) on parental self‐regulation and parent school‐based involvement. Standardized estimates are displayed. between, between‐participant effects; PSRS, Parenting Self‐regulation Scale; PSI, Partners in Education Survey (PIES) Parent School Involvement subscale; w, within‐participant effects. *p < 0.05; **p < 0.01; ***p < 0.001.

As reported in Figure 2a, the slopes of parent school‐based involvement and parental self‐regulation were positively correlated. This suggested that the improvements in parental self‐regulation were positively associated with improvements in parent school‐based involvement. Besides the positive association between intercepts, the parental self‐regulation intercept was negatively associated with the parent school‐based involvement slope, which indicated that higher levels of baseline parental self‐regulation were associated with lesser improvements in parent school‐based involvement. This could represent a potential ceiling effect as highly self‐regulated parents tended to have higher baseline levels of parent school‐based involvement, thus having less room for improvement.

##### 3.3.2.2 RI‐CLPM

As displayed in Figure 2b, parental self‐regulation and parent school‐based involvement were positively associated at the between‐participant level. At the within‐participant level, after accounting for the within participant concurrent correlations and autoregressive regressions, parental self‐regulation at earlier measurement points was found to have a unidirectional, positive association with parent school‐based involvement at successive measurement points, with a large cross‐lagged regression effect. This indicated that improvements in parental self‐regulation following the intervention could contribute to increases in parent school‐based involvement, but not vice versa.

## 4 Discussion

To our knowledge, this is the first study exploring the associations between parental self‐regulation and two dimensions of the home–school partnership, namely parent–teacher communication and parents' involvement in school‐based activities, through mechanism of change analysis. Detailed discussion on the interventions' impact on each of the home–school partnership domains is presented in Part I of this article series (Ma et al. 2026a). The discussion below has a focus on the mechanisms of change, which is the focus of this paper.

### 4.1 Parent–Teacher Communication

Statistically significant improvements in parent–teacher communication were found at post‐intervention and follow‐up assessments. Mechanism of change analyses suggested that both the baseline levels and the rates of change of parent–teacher communication and parental self‐regulation were positively correlated. This is consistent with theoretical frameworks (Epstein 1995; Hoover‐Dempsey et al. 2005) and meta‐analysis (Ma, Tellegen, Hodges, and Sanders 2024). In addition, the findings supported the notion that these associations were bidirectional in nature, such that improvements in parent–teacher communication could be attributed to the increased levels of parental self‐regulation and vice versa. Parents with high levels of parental self‐regulation tended to have more effective communication with teachers, while positive experiences of communicating with teachers further enhanced parents' levels of parental self‐regulation. This bidirectional association is in line with Bandura's theory (Bandura 1997) that self‐efficacy and contextual factors reciprocally influence each other, and also with the findings of a recent cross‐sequential study where the bidirectional associations were found between waves 2 years apart (Ma, Tellegen, and Sanders 2024).

There are various ways in which parental self‐regulation can contribute to the quality of parent–teacher communication. For example, highly efficacious parents tended to be more proactive and autonomous in communicating with teachers about their children's learning and well‐being, and were less likely to avoid such interactions (Harpaz and Grinshtain 2020). Also, in situations where children experience difficulties with learning or behavior, highly efficacious parents were less likely to perceive teachers' communication about these issues as criticism of their parenting abilities (Harpaz and Grinshtain 2020). Moreover, parents with higher levels of parental self‐regulation tended to have better capacity to regulate their emotions and behavior when interacting with their children's teachers (Sanders and Mazzucchelli 2013; Sanders et al. 2019). This capacity could allow parents to be perceived as less threatening to teachers, to feel less threatened by teachers, and to be more constructive and collaborative when working with teachers (Sanders and Mazzucchelli 2013).

### 4.2 Parent School‐Based Involvement

Parents' participation in school‐based activities significantly improved following the intervention and was maintained at the follow‐up assessment. Suggested by findings from the bivariate‐LGCM, higher baseline levels of parental self‐regulation were associated with higher baseline levels of, and less improvements in parent school‐based involvement, suggesting a ceiling effect. The rates of change of these two factors were also positively associated. This is in support of the theoretical frameworks (Hoover‐Dempsey and Sandler 1997), and findings of previous cross‐sectional empirical studies (Ma, Tellegen, Hodges, and Sanders 2024). In addition, subsequent analysis found that an earlier wave of parental self‐regulation was positively and unidirectionally associated with parent school‐based involvement at the successive wave. This is contrary to Bandura's theory of self‐efficacy (Bandura 1997) and the findings of a previous longitudinal study where bidirectional longitudinal associations were found (Choe 2022). However, instead of suggesting the association between parental self‐regulation and parents' participation in school‐based activities is unidirectional, it is more likely that the conservative nature of the analytical approach (i.e., RI‐CLPM) led to insufficient statistical power to detect bidirectional, within participant, cross‐lagged associations (Mulder 2023). This is evident firstly, in the magnitude of the standardized cross‐lagged associations between earlier parent school‐based involvement and later parental self‐regulation that fall under the medium to large cross‐lagged regression effect category proposed by Orth et al. (2024), and secondly, in the close to statistical significant significance values (*p* = 0.063 and 0.071 respectively).

### 4.3 Implications

One significant implication of the present study for school leaders, teaching staff, school counselors, and school psychologists is the importance of recognizing the important role parental self‐regulation plays in parents' participation in children's learning and in their interaction with the school. It is important for schools to recognize that current levels of home–school partnership can be partially determined by parents' current levels of self‐regulation, which may have been shaped by a range of proximal and historical factors, but can also be influenced by ongoing efforts to support parents' capacity to participate in the home–school partnership by promoting self‐regulatory capacity in their parenting role (Ma, Tellegen, and Sanders 2024). It is important to note that one major way to promote parental self‐regulation is through delivering evidence‐based parenting interventions through schools (Sanders et al. 2021, 2014; Sanders and Mazzucchelli 2013). The value of providing universal access to evidence‐based parenting programs through schools is highlighted in this study as it could not only bring benefits for children's social, emotional, and behavioral adjustments (Sanders et al. 2021, 2014), but also have significant spillover benefits for the partnership between a child's home and their school.

### 4.4 Limitations

Despite the significant strengths of the present study, the findings need to be interpreted considering the study limitations. Firstly, due to practicality and feasibility reasons, this study relied on parent self‐report data for all study outcomes. However, self‐report data could be prone to response bias. Secondly, although the sample size is large compared to most evaluation studies for parenting programs (Sanders et al. 2014), the power of the present study in finding significant cross‐lagged regressions remains low as samples with over 1500 participants are usually recommended for research using RI‐CLPM (Mulder 2023). Type II error might be the cause of not finding bidirectional effects for parents' involvement in school‐based activities. Thirdly, this study focused specifically on parental self‐regulation as the hypothesized mechanism of change underlying improvements in the home–school partnership, demographic covariates, other relevant factors (e.g., parenting stress, teacher practices, or school context), and subgroup analyses were not included or conducted as the models were designed to test within‐person longitudinal mechanisms and subgroup sample sizes were insufficient for reliable comparisons. These warrant future research. In addition, the sample characteristics remain typical of most research studies involving parents (e.g., majority were female, highly educated, and from not socially disadvantaged background). The generalizability of the findings to other populations is yet to be determined. Worth noting that past research on evidence‐based parenting programs often found that families from disadvantaged backgrounds tend to benefit as much as families from other backgrounds if they can be effectively recruited and retained in the programs (Buckley et al. 2025; Sanders, Ma, et al. 2024).

## 5 Conclusion

This study examined the positive associations between parental self‐regulation and two dimensions of the home–school partnership, namely parent–teacher communication and parents' involvement in school‐based activities. Through conducting a mechanism of change analysis on data collected from a cluster randomized trial of an evidence‐based parenting program, improvements in the home–school partnership were attributed to post‐intervention improvements in parental self‐regulation. The present findings highlighted the spillover benefits of providing universal access to low‐intensity evidence‐based parenting programs through schools.

## Funding

This research was supported by the Australian Government Department of Education through the Emerging Priorities Program. This funding source did not contribute to the design, execution, analyses, interpretation of the data, or decision to submit results. This research was also partially supported by the Australian Government through the Australian Research Council's Centre of Excellence for Children and Families over the Life Course (Project ID: CE200100025).

## Conflicts of Interest

The Parenting and Family Support Centre is partly funded by royalties stemming from published resources of the Triple P—Positive Parenting Program, which is developed and owned by The University of Queensland (UQ). Royalties are also distributed to the Faculty of Health, Medicine, and Behavioural Sciences at UQ and contributory authors of published Triple P resources. Triple P International (TPI) Pty Ltd. is a private company licensed by Uniquest Pty Ltd. on behalf of UQ to publish and disseminate Triple P worldwide. The authors of this report have no share or ownership of TPI. TPI had no involvement in the study design or analysis or interpretation of data. Prof Sanders receives royalties and/or consultation fees from TPI. Dr. Hodges may receive royalties in the future. Dr. Ma, Dr. Tellegen, and Dr. Hodges are employees at UQ.

## Data Availability

The data that support the findings of this study are available on request from the corresponding author. The data are not publicly available due to privacy or ethical restrictions.
